# Maternal Feeding Styles and Child Appetitive Traits: Direction of Effects in Hispanic Families With Low Incomes

**DOI:** 10.3389/fpubh.2022.871923

**Published:** 2022-06-02

**Authors:** Maria A. Papaioannou, Nilda Micheli, Thomas G. Power, Teresia M. O'Connor, Jennifer Orlet Fisher, Sheryl O. Hughes

**Affiliations:** ^1^USDA/ARS Children's Nutrition Research Center, Department of Pediatrics, Baylor College of Medicine, Houston, TX, United States; ^2^Department of Human Development, Washington State University, Pullman, WA, United States; ^3^Center for Obesity Research and Education, Department of Social and Behavioral Sciences, College of Public Health, Temple University, Philadelphia, PA, United States

**Keywords:** Hispanic families, feeding styles, bi-directional effects, cross-lagged panel analysis, child appetitive traits

## Abstract

Feeding styles of parents have been associated with dietary quality/intake and weight outcomes; however, much of the research to date has been cross sectional and the direction of influence unclear. This prospective longitudinal study evaluated the direction of effects between feeding styles and child appetitive traits over time in a sample of 129 Hispanic parent/child dyads that participated in a larger study. Data analyzed for the current study were collected when the children were 4–5 years old and again at ages 7–9 years. Parents (all mothers) reported on their feeding styles and children's appetitive traits using well-established questionnaires. Cross-lagged panel analyses were used to examine the direction of effects. Fully adjusted models revealed that a number of children's appetitive traits at baseline predicted later feeding styles. A bi-directional relationship was found between authoritarian feeding and satiety responsiveness such that higher satiety responsiveness was associated with authoritarian feeding and vice versa. Lower satiety responsiveness was associated with indulgent feeding, whereas higher food responsiveness was associated with authoritarian feeding. Results show preliminary evidence that children's appetitive traits may shape mothers' approach to child feeding. There is also preliminary support for the protective role of an authoritarian feeding style in the self-regulatory processes around child appetitive traits among this population of Hispanic families with low-income levels. These results warrant continued research given that other studies have shown beneficial outcomes for authoritarian feeding among ethnically diverse families with low incomes.

## Introduction

Parental feeding plays a major role in the development of child eating including food preferences, appetitive traits, and dietary quality/intake (1–6). Parental feeding influences what, when, and how much children eat and has been linked to the above mentioned child eating behaviors (7) as well as childhood obesity (2, 4). Parental feeding includes both goal oriented feeding practices such as restriction and pressure to eat as well as feeding styles, the broader, more general approach parents use to socialize their children around eating (8). The concept of feeding style includes the emotional climate created between parents and their children during eating events (8, 9). Feeding styles are thought to influence appetite self-regulation in children. Appetite self-regulation involves a wide range of trait-like behaviors that reflect biological bottom-up and cognitive top-down aspects of eating that are reflected in hunger and satiety responses (10). Appetite self-regulation shapes the quality of dietary intake and quantity of food eaten by children (i.e., portion sizes). Similar to general self-regulation in children, bottom-up processes are thought to involve biological drives toward food motivation and avoidance, whereas top-down processes are thought to involve cognitive appraisal (10).

Feeding styles have been consistently associated with child eating and weight (9). For example, the authoritative feeding style has been associated with lower intake of snack foods (11) and better diet quality of meals served to and consumed by children at dinnertime (12). In contrast, the indulgent feeding style has been associated with higher intake of energy dense snacks (11), lower intake of vegetables, dairy, and fruit (13), and larger portion sizes selected (14). Similarly, the uninvolved feeding style has been associated with less healthy outcomes such as lower child intake of fruit and vegetables (13). Overall, consistent evidence has shown that the indulgent feeding style is associated with more problematic child eating—more energy dense foods, greater child self-served portion sizes, and higher weight status across cross-sectional and longitudinal studies (9).

Despite the plethora of studies on feeding practices and styles, child food preferences, appetitive traits, and dietary quality/intake, and weight, many of these studies use cross-sectional designs prohibiting causal inferences and/or the examination of the direction of influence (i.e., parent, child, or both). Developmental scientists typically emphasize a bi-directional relationship between the parent and child (15); however, the common view of the feeding relationship is unidirectional, emphasizing parental behaviors directed toward the child. This perspective is problematic as it does not allow for child characteristics that may influence feeding interactions, such as mother's response to the highly food motivated child (16, 17). It is becoming increasingly apparent in the feeding literature that the parent-child relationship is likely reciprocal (18–22).

Only a handful of studies (*n* = 7) have evaluated bi-directional relationships of parental feeding and various child eating behaviors. Furthermore, all of those studies focused on goal-directed feeding practices. Specifically, five of these studies found bi-directional effects with feeding practices predicting child food responsiveness, emotional overeating, and eating large amounts of food and vice versa (18–22). In contrast, two studies found effects only from feeding practices to child appetitive traits, such as eating in the absence of hunger (23) and emotional overeating, food responsiveness, and enjoyment of food (24). For the most part, these studies targeted highly coercive practices (i.e., restriction, pressure to eat, food as a reward, and/or similar constructs) and a few structured practices (i.e., monitoring and family meals). For example, instrumental feeding (i.e., using food as a reward) was found to have a positive bi-directional association with child food responsiveness (18) and emotional overeating (18, 21). Monitoring and pressure to eat were found to have a negative bi-directional association with children eating large amounts of food (19). Monitoring also showed a negative bi-directional relationship with food refusal whereas pressure to eat showed a positive bi-directional relationship with the same construct (19).

Findings from these studies give rise to the possibility of a complex bi-directional relationship. However, only highly controlling feeding practices (e.g., restriction, pressure to eat, and using food as a reward) were targeted in these studies. The focus on goal-oriented behaviors may be too narrow when attempting to understand the complex mechanisms leading to obesity related health outcomes in children. Examining a more global approach to feeding may allow for the inclusion of aspects of the parent-child feeding relationship that are beyond the measurement of specific type feeding practices. Unfortunately, no studies to date have examined bi-directional effects using the feeding styles construct. Examining feeding styles is important as it represents a more consistent construct over time and across the various contexts that parent-child eating occasions occur (25).

Therefore, we used cross-lagged panel analyses to examine parent-reported feeding styles and child appetitive traits across two time periods (i.e., child ages 4–5 and 7–9), using longitudinal data from a previous study of how child appetitive traits develop among a Hispanic sample of families with low-income levels (26, 27). We included child appetitive traits that have shown consistent relationships with weight in previous studies (28). Specifically, satiety responsiveness has been negatively, and food responsiveness and emotional overeating have been positively related to child weight.

**Figure 1:**
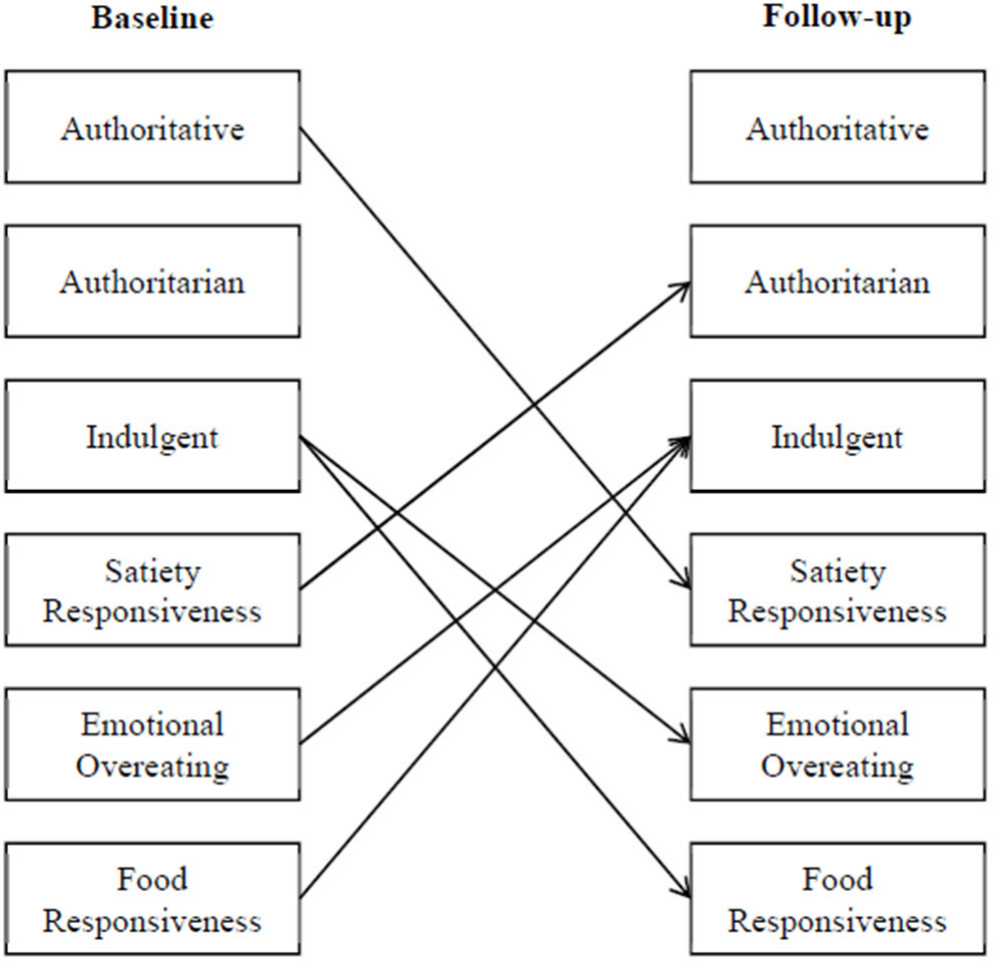
Conceptual model depicting hypothesized paths.

The aim of this study was to prospectively evaluate the direction of influence between feeding styles and child appetitive traits over a 3-year period in early childhood among a diverse sample of children with low-income backgrounds. In examining the influence from child appetitive traits (ages 4–5) to feeding styles (ages 7–9), we hypothesized that children who were satiety responsive (food avoidant) would have mothers who reported an authoritarian feeding style. In contrast, we predicted that children who were characterized by emotional overeating and food responsiveness (food approaching) would have mothers who reported an indulgent feeding style. In examining the influence from feeding to child appetitive traits, we predicted that mothers who exhibited an authoritative feeding style would have children who were more satiety responsive (ability to cease consumption in response to internal signals) as these mothers are more autonomy supportive. We predicted that mothers who exhibited an indulgent feeding style would have children who were more likely to be food approaching (emotional overeating and food responsive). Figure 1 shows the hypothesized paths from feeding styles to child appetitive traits and vice versa in a conceptual model. Results from this study provide a broader view of the feeding and eating dynamics between parents and their children overtime by clarifying the directional influences of their interactions.

## Methods

### Participants

Participants in the current study were 129 Hispanic parents and their children enrolled in Head Start who participated in a previous longitudinal study (*n* = 187) of how child appetitive traits develop in families with low-income levels (27, 29). All participating parents were mothers; thus, parents will be referred to as mothers hereafter. A convenience sample of mothers and their children were recruited from Head Start districts in a large urban city in the southern part of the United States beginning in 2011. Eligibility criteria were mothers self-identifying as Hispanic (either English or Spanish speaking) and their child attending Head Start (ages 4 or 5) at the time of recruitment. Exclusion criteria included mothers and/or children with extensive dietary restrictions (e.g., those with diabetes, food allergies, or on special diets) and children with developmental problems limiting their ability to perform study tasks (e.g., autism and significant developmental delays). If issues of eligibility arose, a pediatrician (co-investigator) reviewed the dietary restrictions and/or developmental delay diagnoses to determine participation in the study. The study was reviewed and approved by the Institutional Review Board at the Baylor College of Medicine (ethics approval number H-26796). The purpose of the study was explained to mothers in their language of choice (i.e., English or Spanish). Written consent for their participation as well as child verbal assent were obtained. Consenting procedures took place before study activities began at both baseline and the follow-up.

The original sample of 187 mother-child dyads were recruited at baseline for the larger longitudinal study (26). Approximately 18 months after baseline (*M* = 18.39, *SD* = 1.58), assessments were conducted on 144 dyads. Finally, ~24 months after the follow-up (*M* = 23.6, *SD* = 6.54), assessments were conducted on 129 dyads. More information on recruitment and retention can be found in a previous publication (27). Data from the first follow-up were not included in the present study to reduce the number of analyses and because individual differences in child appetitive traits were relatively stable between baseline and the first follow-up, mean *r* = 0.53. Less stability was observed between baseline and the second follow-up (referred to as “follow-up” henceforth), mean *r* = 36. A total of 129 mothers and their children had data on all variables for analyses in the current study. Children's ages at baseline and the follow-up assessment were *M* = 4.76 (*SD* = 0.46) and *M* = 8.34 (*SD* = 0.71), respectively.

Presented in Table 1 are the demographics on mothers whose data were analyzed for this study—a subsample of the 187 mother-child dyads. Mothers were an average of 31.55 years old (*SD* = 6.6), and most were homemakers (79.1%), married (58.9%), and either born in Mexico (63.5%) or Central America (17.9%). Mothers showed a range of educational status (ranging from 6th grade to beyond college graduate). About half of the children were female and about half had a healthy weight status (1.6% were in the underweight category). Twenty-two and one half percent were in the overweight category and 27.1% were in the obese category. Combined, these percentages are higher than the 30% of 2- to 5-year-old Hispanic children in the United States who are considered overweight or obese (30). We expected higher levels of children having overweight and obesity in our sample compared to those in the general population because our sample was urban with low incomes from the southern region of the United States. Moreover, our participants were toward the upper end of the 2- to 5-year-old age range; since the prevalence of overweight and obesity increases with age, we expected levels higher than average across our age range. No significant differences were found on demographic variables between the 129 mothers and children who had data at both baseline and follow-up and the initial sample of 187 mothers and children (Table 1). Participants in this study were not necessarily representative of the Hispanic population in the United States but may be representative of those in this geographical area.

**Table 1:** Characteristics of the sample at baseline.

**Characteristics**	**All participants, *M* (SD) or % (*n* = 129)**
Parent gender—female	100.0
Child gender—female	53.5
Parent age, mean in years (SD)	31.55 (6.60)
Child age, mean in years (SD)	4.76 (0.46)
**Education of parent**	
Less than high school diploma	38.0
High school diploma or equivalent	24.0
Some college or more	38.0
Employment status, currently employed	20.9
**Marital status**	
Married	58.9
Never married	14.0
Widowed, separated, divorced	27.1
**Parent immigrant status**	
Born in the U.S.	17.8
Born in Mexico	63.5
Born in Central America	17.9
Born in Cuba	0.8
**Child immigrant status**	
Born in the U.S.	96.9
**Child BMI categories**	
Underweight (<5th percentile)	1.6
Healthy (5th to <85th percentile)	48.8
Overweight (85th to <95th percentile)	22.5
Obese (>95th percentile)	27.1

### Measures

Questionnaires used in the study were translated into Spanish using standardized procedures and have shown reliability and validity in Hispanic samples—Caregiver's Feeding Styles Questionnaire (8, 31, 32), and Children's Eating Behavior Questionnaire (33). Demographic information was obtained including birth dates (parent and child), ethnicity, race, gender, education, marital status, employment status, and immigrant status.

#### Caregiver's Feeding Styles Questionnaire

Mothers reported on their feeding style using the CFSQ (8), which is designed to assess feeding styles in families with low-income levels and has been used successfully with Hispanic families (3). Parents responded to 19 items using a 5-point response scale ranging from 1 = Never to 5 = Always. Dimensions of demandingness (i.e., how much parents encourage eating during eating episodes) and responsiveness (i.e., how parents encourage eating; the level of nurturance parents use in directing child eating) were calculated using seven child-centered items (e.g., asking questions, providing reasons, and allowing choice) and twelve parent-centered items (e.g., using food as a reward, hurrying the child, and spoon-feeding the child) (8). A cross-classification of high and low scores on these dimensions translates into four feeding styles: authoritative (high responsiveness, high demandingness), authoritarian (low responsiveness, high demandingness), indulgent (high responsiveness, low demandingness), and uninvolved (low responsiveness, low demandingness). Because children's eating becomes more autonomous with increasing age (and parental demands decrease), different median splits were used for demandingness at baseline and the follow-up (based on median scores for demandingness at each time point, 3.05 and 2.53, respectively). The corresponding medians for responsiveness, which did not change with age, were 1.19 and 1.22. A more detailed discussion of the scoring procedure can be found elsewhere (8). Evidence of test-retest reliability, internal consistency, convergent and predictive validity has been demonstrated (3). The CFSQ has been validated with direct observation of parent/child interactions during mealtimes (31). The CFSQ has been used successfully in studies of parents with children in elementary school (34–36) as well as with younger ages (8, 31). Coefficient alphas for child-centered and parent-centered items were 0.67 and 0.84, respectively.

#### Children's Eating Behavior Questionnaire

Mothers reported on their child's eating by completing the CEBQ which has established factor structure, test-retest reliability, and internal consistency (37). The CEBQ measures eight dimensions of eating including four subscales assessing food approach (food responsiveness, emotional overeating, enjoyment of food, desire to drink) and four subscales assessing food avoidance (satiety responsiveness, slowness in eating, emotional under-eating, and food fussiness) (37). To minimize the number of variables in the analyses, three subscales were used: two food approach subscales of food responsiveness (e.g., “My child is always asking for food”) and emotional overeating (e.g., “My child eats more when worried”); and one food avoidant subscale of satiety responsiveness (e.g., “My child gets full before his/her meal is finished”). These subscales were chosen because they reflect individual differences in child appetitive traits and have been linked prospectively to parental feeding and/or child weight in previous studies (28) (see Section Data Analyses for further clarification). Coefficient alphas in the current sample were 0.80 for food responsiveness, 0.70 for emotional overeating, and 0.68 for satiety responsiveness.

#### Bi-Dimensional Acculturation Scale

The BAS was used to measure mothers' acculturation to the U.S. culture (38). It consists of three subscales: language use with 6 items (e.g., How often do you speak English?), language proficiency with 12 items (e.g., How well do you read in English?), and electronic media with 6 items (e.g., How often do you watch television programs in English?). As originally described by Marin and Gamba (38), the three subscales were combined to create a Spanish domain and an English domain, which had high internal consistency in the original study (alpha of 0.90 and 0.96, respectively). Cronbach's alphas were acceptable in this sample: Spanish and English domains (alpha of 0.92 and 0.97, respectively). Because there was very little variability in the Spanish domain score (almost 90% of the participants had a score of three or above on a scale of one to four), we used only the English domain score in the analyses.

#### Anthropometrics

Trained research staff took child height and weight measurements following a standard protocol (39). Children were measured twice with no shoes and wearing light clothing using a stadiometer (Seca model 214, Seca, China) and an electronic self-calibrating digital scale (Health-O-Meter model 752KL, Health O Meter, China). Measurements were recorded to the nearest 0.1 kg (weight) and 0.1 cm (height). Using the Centers for Disease Control and Prevention Reference Standards, age- and gender-specific Body Mass Index (BMI) standardized scores (BMI *z*-score) were calculated (40) and children were categorized as underweight (BMI < 5th percentile), healthy weight (BMI ≥5th to <85th percentile), overweight (BMI ≥85th to <95th percentile), or obese (BMI ≥ 95th percentile).

### Data Analyses

Data were analyzed using AMOS (version 27) and *p* < 0.05 was used in all analyses. To address missing data for subscale scores, if 25% or less of the items on a given subscale were blank, the subscale score was calculated by computing the mean of the non-missing items. If more than 25% of the items were blank, the score on the given subscale was considered missing. The cross-lagged analysis was conducted using six primary variables at each time point and five controls (child gender, child BMI *z*-score at baseline, maternal education, maternal acculturation, and maternal BMI at baseline). The six primary variables were three dummy coded variables to represent the four feeding styles (with uninvolved feeding as the reference group) and the three child appetitive traits as measured by the satiety responsiveness, emotional overeating, and food responsiveness subscales of the CEBQ.

We chose authoritative, authoritarian, and indulgent feeding styles because of their consistent associations with child weight status (positively or negatively) in previous studies of general parenting or feeding styles (3). We chose appetitive traits that reflect individual differences in child appetite self-regulation. Specifically, satiety responsiveness is a measure of better appetite self-regulation, while food responsiveness and emotional overeating are measures of poorer appetite self-regulation. Other CEBQ subscales measure the construct less directly (i.e., slowness in eating, desire to drink, and enjoyment of food).

**Figure 2:**
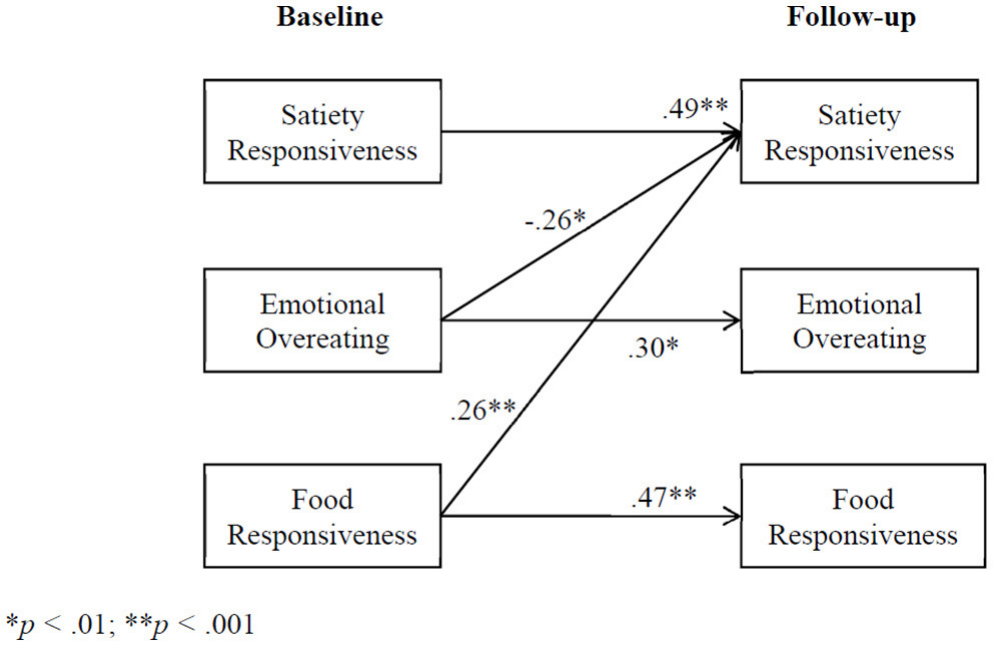
Cross-lagged panel analysis: child appetitive traits to child appetitive traits (unstandardized B weights). *p < 0.01; **p < 0.001.

**Figure 3:**
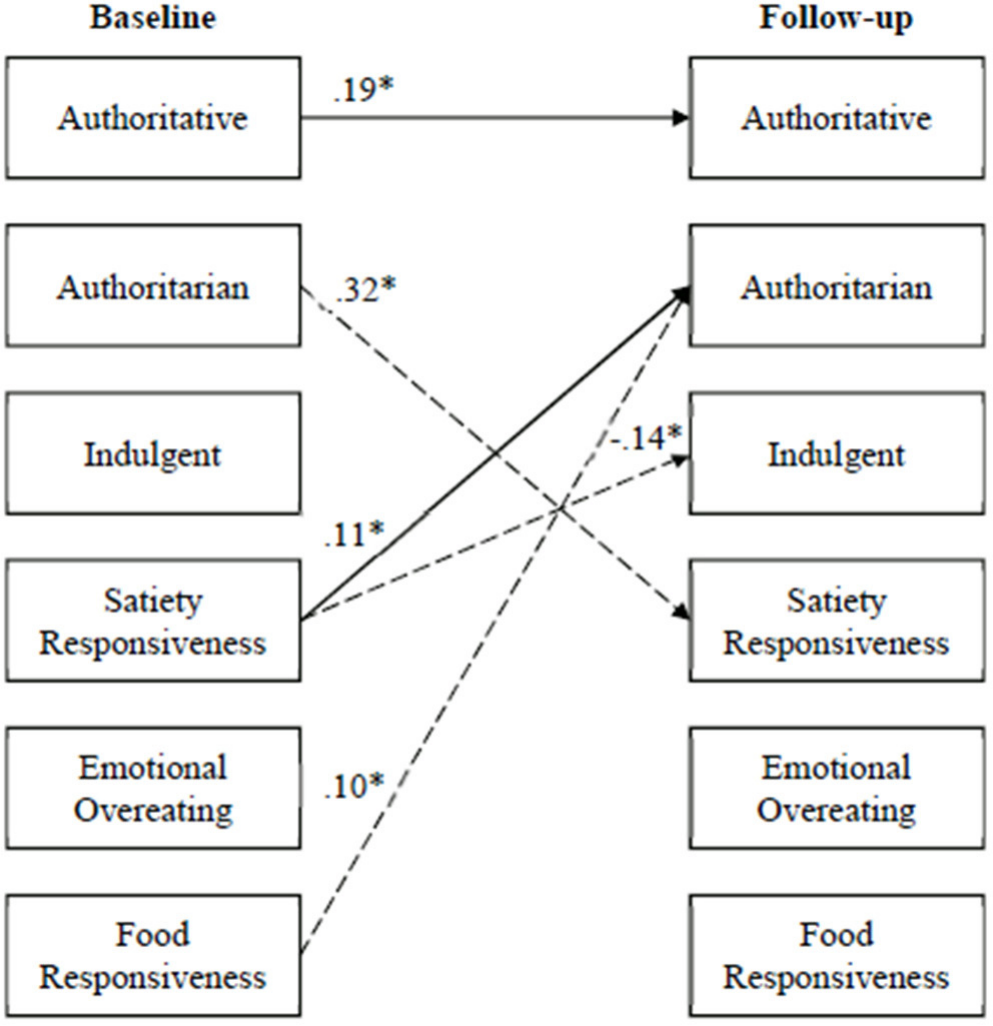
Cross-lagged panel analysis: parental feeding styles to child appetitive traits and vice-versa (unstandardized B weights). Sold lines represent hypothesized effects; dotted lines represent effects not hypothesized. *p < 0.05.

Model fit was determined by examining the chi square (n.s.), the CFI (≥0.95), and the RMSEA (<0.06) (41). Because feeding style (a single construct) was represented by three dummy coded variables, the errors for these variables were allowed to correlate. Unstandardized B weights are presented in Table 2 and Figures 2, 3 because standardized regression weights with dichotomous dependent variables are hard to interpret. Full information maximum likelihood (FIML) was used to estimate missing values.

**Table 2:** Results of cross-lag panel analyses predicting follow-up variables from baseline variables (unstandardized B weights).

	**Follow-up child appetitive traits**			**Follow-up parent feeding style**		
**Baseline predictor**	**Satiety responsiveness**	**Emotional overeating**	**Food responsiveness**	**Authoritative feeding style**	**Authoritarian feeding style**	**Indulgent feeding style**
Satiety responsiveness	0.49^***^	0.12	0.18	0.02	0.11^*^	−0.14^*^
Emotional overeating	−0.26^**^	0.30^**^	−0.06	−0.06	−0.08	0.16
Food responsiveness	0.26^***^	0.03	0.47^***^	0.02	0.10^*^	−0.12
Authoritative feeding style	0.28	0.20	−0.02	0.19^*^	0.05	−0.12
Authoritarian feeding style	0.32^*^	0.12	−0.11	0.15	0.18	−0.20
Indulgent feeding style	0.23	0.18	−0.05	0.05	0.03	0.10
*R* ^2^	0.32	0.17	0.20	0.07	0.11	0.16

* *p < 0.05,*

** *p < 0.01,*

*** *p < 0.001*.

## Results

The results of the cross-lagged panel analyses are presented in Table 2 and Figures 2, 3. To achieve model fit, the error terms between emotional overeating and food responsiveness at follow-up were allowed to correlate, standardized *B* = 0.67, *p* < 0.001. The cross-lagged model showed excellent fit, $X_{\left(11\right)}^{2}$ = 12.35, n.s., *CFI* = 0.998, *RMSEA* = 0.026. As shown by the significance (*p* < 0.05) of the unstandardized B weights in the table, only the authoritative feeding style was significantly stable over time; however, all three child appetitive trait variables were significantly stable. In addition to the stability findings, in two instances, child appetitive traits at baseline significantly predicted child appetitive traits at follow-up (Figure 2). Specifically, emotional overeating at baseline negatively predicted satiety responsiveness at follow-up, and food responsiveness at baseline positively predicted satiety responsiveness at follow-up. As shown in Figure 3, three “child direction of effects” associations were seen where child appetitive traits at baseline predicted parental feeding styles at follow-up: (1) satiety responsiveness at baseline positively predicted authoritarian feeding at follow-up, (2) satiety responsiveness at baseline negatively predicted indulgent feeding at follow-up, and (3) food responsiveness at baseline positively predicted authoritarian feeding at follow-up. Only one “parent direction of effects” association was significant where feeding styles at baseline predicted child appetitive traits at follow-up: authoritarian feeding at baseline positively predicted satiety responsiveness at follow-up.

## Discussion

The current study examined the direction of influence between parent-reported feeding styles and child appetitive traits in a sample of Hispanic mothers from low-income backgrounds and their children. Only a few studies have found bi-directional associations between parental feeding and child outcomes (18–22); however, to our knowledge, this is the first examination of such associations between parental feeding styles and child appetitive traits over time. The results of this study provide initial evidence supporting the premise that children's appetitive traits influence how mothers parent their children around eating. These findings are in contrast to much of the current literature that views parent-child eating under the lens that child appetitive traits are in response to parental feeding directives—despite much of the literature being based on cross-sectional data. Specific to this study, of the six paths hypothesized in the analyses (three paths from feeding to appetitive traits and three from appetitive traits to feeding), only one path from feeding to appetitive traits was significant (from the authoritarian feeding style to satiety responsiveness). In contrast, three paths were significant from child appetitive traits to feeding (satiety responsiveness to authoritarian feeding, satiety responsiveness to indulgent feeding, and food responsiveness to authoritarian feeding).

As predicted, children who were higher in satiety responsiveness at baseline had mothers who were later categorized as authoritarian. Authoritarian feeding style and children who are satiety responsive. This was the only bi-directional relationship found in the study. Although our main findings suggest that the way parents approach feeding their children is guided by child eating characteristics (e.g., food responsive and satiety responsive), this study also provides evidence of a bi-directional relationship where parenting/feeding shapes children's eating behavior in some instances. Additionally, it has been suggested that parenting characterized by high demandingness and warmth but low in autonomy granting, in general, may be a protective behavior in Hispanic families with low-income levels (45). These families may perceive highly demanding parenting behaviors as being involved rather than intrusive. Our findings are consistent with the premise of authoritarian parenting in the feeding context as a protective behavior in Hispanic families (46) which may extend to child appetite self-regulation.

The potentially protective functions of control in the feeding literature mirror findings in general parenting such that types of control associated with negative child outcomes in non-Hispanic samples often show weaker, non-significant, or positive relationships in Hispanic samples (42, 43). Because Hispanic mothers sometimes show high levels of control, certain controlling interactions may not have a negative impact because they are a way through which Hispanic mothers show involvement and caring with their children (44). This interpretation is consistent with the concept of protective parenting in Hispanic families—characterized by high demandingness, high responsiveness, and low autonomy granting (45). High levels of control exhibited by Hispanic parents, rooted in the values of familism and respect for authority, may support healthful child development (45) and be protective against childhood obesity as well.

It was hypothesized that children who were characterized by food approach traits of emotional overeating and food responsiveness would have mothers who reported an indulgent feeding style. Results from this study did not support these hypotheses. This lack of association is surprising as previous studies have shown children who exhibit food approach type behaviors have parents who exhibit higher levels of emotional feeding and use food as a reward (24, 46, 47). It was expected that indulgent feeders may reinforce early emotional eating by not setting limits and letting children eat as much as they want. The previous studies cited above were conducted with predominantly white samples. There is a need for qualitative studies of indulgent feeding among Hispanics with low incomes to better understand the construct within this cultural group. Along the same lines, lower satiety responsiveness at baseline was associated with indulgent feeding at follow-up. Given that satiety responsiveness is considered a food avoidant trait among children, it is not surprising that lower food avoidance was associated with a feeding style characterized by not setting limits and allowing excess food intake.

The finding of higher food responsiveness and authoritarian feeding is supported by previous research showing associations between coercive control type practices (i.e., restriction; threats and bribes) and food approach traits such as food responsiveness (20–22, 24). Authoritarian feeders have been observed to exhibit coercive control practices through direct observation of family meals (8, 31). It makes sense that children who exhibit higher food approach traits would have parents who are authoritarian demonstrating coercive control type practices in an effort to restrict children's intake.

Regarding the stability of the child appetitive traits in the current study, as expected, child appetitive traits (as measured by the CEBQ) were somewhat stable overtime from child ages 4–5 to 7–9 years. This finding is in line with literature showing that child appetitive traits may have a genetic component and that children are born with tendencies toward food avoidance or food approach (48–51). This finding has been demonstrated in several other studies (19, 20, 52–54).

Findings should be considered in light of the limitations. Only one ethnic group was included in the study. Furthermore, with the exception of child height and weight that was objectively measured, parental feeding styles and child appetitive traits were assessed using parent-reported questionnaires. Social desirability has been implicated as a bias in parent-report of their own behavior and that of others (e.g., their children) (55). However, one strength is the wide use of the chosen questionnaires in the overall feeding literature (9, 28) and the validation of the CFSQ with direct observation (31). Another strength is the use of a longitudinal design which allowed for examination of the direction of influence of feeding styles and child appetitive traits overtime from preschool to middle childhood within the Hispanic cultural group.

In conclusion, based on the findings of this study, there is preliminary evidence showing that children's appetitive traits may shape mothers' approach to child feeding. Furthermore, there is also preliminary support for the protective role of an authoritarian feeding style in child self-regulatory processes around eating among this population of Hispanic families. Satiety responsiveness which was prospectively associated with authoritarian feeding over time has been consistently linked to lower child weight across multiple previous studies (28). Results need to be replicated in a larger sample and understood within the parenting culture of this ethnic group. Specifically, additional studies are needed to understand the potential protective role of the highly demanding authoritarian feeding style on more healthful child outcomes among Hispanic and other ethnically diverse families with low incomes.

Overall, most studies examining parental feeding and child appetitive traits are cross-sectional and those that are longitudinal assume that the direction of effects is from the parent to the child. Findings from this longitudinal study support future efforts examining how child characteristics may shape the parent-child dynamic as it relates to childhood obesity. Targeting this dynamic in childhood obesity prevention programs will foster better child outcomes and support public health efforts to reduce obesity among children.

## Data Availability Statement

The raw data supporting the conclusions of this article will be made available by the authors, without undue reservation.

## Ethics Statement

The studies involving human participants were reviewed and approved by Institutional Review Board at the Baylor College of Medicine. Written informed consent to participate in this study was provided by the participants' legal guardian/next of kin.

## Author Contributions

SH and TP conceived the research question and designed the study. MP, TP, and SH drafted the manuscript. SH oversaw all data collection. TP ran the data analyses. TO'C and JF assisted with the design of the study and provided comments on the manuscript. NM coordinated all data collection and provided comments on the manuscript. All authors were involved in writing the paper and had final approval of the submitted and published versions.

## Funding

This research was supported by funds from the National Institute of Child Health and Human Development (Grant R01 HD062567). This work is also a publication of the US Department of Agriculture/Agricultural Research Service (USDA/ARS) Children's Nutrition Research Center, Department of Pediatrics, Baylor College of Medicine (Houston, TX) funded in part by the USDA/ARS (Cooperative Agreement 6250-51000).

## Author Disclaimer

The contents of this publication do not necessarily reflect the views or policies of the USDA, nor does mention of trade names, commercial products, or organizations imply endorsement from the US government.

## Conflict of Interest

The authors declare that the research was conducted in the absence of any commercial or financial relationships that could be construed as a potential conflict of interest.

## Publisher's Note

All claims expressed in this article are solely those of the authors and do not necessarily represent those of their affiliated organizations, or those of the publisher, the editors and the reviewers. Any product that may be evaluated in this article, or claim that may be made by its manufacturer, is not guaranteed or endorsed by the publisher.
